# Acoustic Emission Monitoring of Multicell Reinforced Concrete Box Girders Subjected to Torsion

**DOI:** 10.1155/2014/567619

**Published:** 2014-08-11

**Authors:** Marya Bagherifaez, Arash Behnia, Abeer Aqeel Majeed, Chai Hwa Kian

**Affiliations:** Department of Civil Engineering, Engineering Faculty, University of Malaya, 50603 Kuala Lumpur, Malaysia

## Abstract

Reinforced concrete (RC) box girders are a common structural member for road bridges in modern construction. The hollow cross-section of a box girder is ideal in carrying eccentric loads or torques introduced by skew supports. This study employed acoustic emission (AE) monitoring on multicell RC box girder specimens subjected to laboratory-based torsion loading. Three multicell box girder specimens with different cross-sections were tested. The aim is to acquire AE analysis data indicative for characterizing torsion fracture in the box girders. It was demonstrated through appropriate parametric analysis that the AE technique could be utilized to effectively classify fracture developed in the specimens for describing their mechanical behavior under torsion. AE events localization was presented to illustrate the trend of crack and damage propagation in different stages of fracture. It could be observed that spiral-like patterns of crack were captured through AE damage localization system and damage was quantified successfully in different stages of fracture by using smoothed *b*-value analysis.

## 1. Introduction

The RC civil infrastructures will deteriorate and degrade during their service due to loading and external environmental exposure conditions. Malfunction of these structures may cause catastrophic events to the society. Therefore, frequent monitoring should be conducted to estimate the health condition of these structures [1, 2] for strategic maintenance purposes. Various testing methods including both the destructive testing and nondestructive testing (NDT) techniques with distinct working principles can be adopted to monitor and assess the integrity of structures. The NDT methods are often more desirable as they do not cause damage to the structure under assessment. The Impact Echo method, ultrasonic pulse velocity (UPV) method, infrared thermography, and acoustic emission (AE) technique are some of the popular NDT methods capable of detecting and evaluating structural deficiencies [3–5]. AE technique involving wave analysis has been widely used in the field of civil and mechanical engineering for structural health monitoring (SHM) [6–13]. One distinct advantage of using AE technique compared to other NDT techniques is that the position of developing crack in the measured structure can be determined, in addition to its nature of implementation, where no interference to the structure under service is instigated. Through AE data analysis, classification and crack direction can be calculated through different approaches such as *b*-value analysis [14–19]. In this current study, the AE technique was utilized to monitor RC multicell box girders under torsion loading. Analysis was carried out on the recorded AE data to provide torsion damage evaluation of the girders in both qualitative and quantitative manners.

## 2. Multicell Box Girder

**Figure 1:**
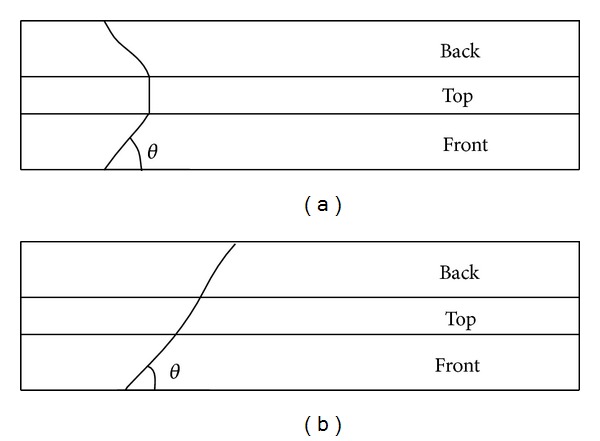
(a) Shear and (b) torsional cracking [21].

The transversal torsional deflection and bending torsional deformation of bridge girder due to transversal seismic action or lateral wind force may have great effect on bridge behavior [20]. For this reason, many researchers have paid much attention to the studies of torsional capacity, bending torque coupling effect, and loss of stability due to lateral inclination. A concrete structure loaded in torsion may be compared to a concrete structure affected by shear. Cracks that develop in concrete due to torsional loading usually follow a mechanism resembling that of concrete under shear loading. The main difference between shear and torsional cracking is that the torsional crack usually forms spiral-like patterns (Figure 1). The crack opens when the principal strain exceeds the tensile strength of the concrete [21].

A significant number of RC bridges have multiple box cells. This type of bridge is aesthetically pleasing due to the closed bottom slab and its shallow depth. This is especially true when curved or sloped exterior webs cantilever overhangs are used, which gives a compact appearance. From the structural point of view, the closed cross-section of a multicell box is ideal to carry eccentric loads or torques introduced by skew supports. The high internal statical indeterminacy allows excellent transverse distribution of reactions and applied loads even without intermediate transverse diaphragms. The RC multicell box girder bridges generally feature substantial overload capacities owing to the availability of force redistribution throughout the structure. However, it is also noted that, due to this complex structural system, it becomes difficult for design engineer to assess the exact load transfer within the structure [22]. Numerous experimental and theoretical investigations have been carried out to clarify the complex behavior of multicellular structures. A summary of experimental and analytical studies on multicell box girder bridges has been given by Scordelis [23]. However more investigation is needed particularly on the fractural mechanism of the multicell girders.

## 3. Acoustic Emission (AE) Technique

Acoustic emission is defined as the transient elastic wave generated by the rapid release of energy from a localized source or sources within a material. The plastic energy propagates as a stress wave (AE event) in the structure and excites a sensitive piezoelectric-transducer. As the stress in the material is raised, a high amount of emissions is generated. Sources of AE can be moving dislocations, crack onset, growth and propagation of crack, fiber breaks, disbands, plastic deformation, and so on caused by various external loading conditions [24]. In contrast to most NDT methods, the energy that converts to AE signal comes from the material itself [25]. Another distinguishable characteristic of AE is its ability in locating structural discontinuities and flaws without having to conduct a point by point search over the entire surface of the structure. These unique characteristics give AE technique huge potential in becoming a promising NDT technique. Therefore there is a need to conduct more investigations and to develop the application of AE technique in structural assessment. Most of the investigations into the assessment of signal characteristics of AE are accomplished for RC beams under the flexural forces. Some aspects of the structural behavior monitoring of RC need to be more investigated further and so far developed members are not investigated thoroughly, such as AE signal characteristics of an RC beam under torsion. In fact, physically, there is no difference between torque and bending. They are exactly the same in a mathematically defined sense. They are both a cross product of a radius vector from an origin to a point of application with the force applied at that point of application. But mechanically they are very different. Torque generates a twisting deflection of an element and generates pure shear or to somehow indirect tension. Moments, on the other hand, generate a curvature or bend in structural elements. Moments generate normal stresses. However, since they have different mechanical effects on the structure then they may cause different stress waves, resulting in different shapes of waveform and AE results [26]. The most crucial point is associated with the fracture behavior which is the most influential issue on the AE results. It should be noted that in bending case the specimen will be imposed to the normal stress and the failure will trigger by mode 1 of fracture and in most cases the specimens will experience shear failure in ultimate condition which is mode 2. But for pure torsion the failure begins in mode 3 and the crack will extend in mode 3 of fracture. In the final failure stage, while shear crack is extending and the crack width is widening, the crack width widening is due to the existing normal stress in the shear plane. Therefore, in the fracture process under pure torsion although it starts with mode 3 of fracture, it will be accompanied with mode 1 in final failure. However, it can be observed that for bending and torque moment we have two different mechanical behaviors and fracture modes which result in different stress elastic waves and AE signals [27]. Particularly, this difference can be found in parameters like rise time, RA, and average frequency since they have been using discrete mode 1 and mode of fractures in existing literature.

The signal characteristic parameters are used to evaluate the grade of damage as well as to identify the nature of damage. The parameter analysis of an AE event evaluates and correlates AE features such as counts, amplitude, rise time, and hits. The classification of these parameters drives the investigator towards correlating AE with its source. The damage level of any kind of structural member can be evaluated qualitatively and quantitatively. AE hits and cumulative signal strength (CSS) are two parameters that can be used for qualitative analysis. The three methods commonly used in concrete structures for quantitative analysis are intensity analysis (IA), *b*-value, and Felicity and Calm ratio [28].

### 3.1. Cumulative Signal Strength (CSS)

An increase in rate of recorded AE indicates an increase in the rate of crack propagation. Therefore the total number of AE hits as function of time is an important test parameter. However there are more valuable parameters such as cumulative signal strength.

Signal strength is defined as the integral of the rectified voltage signal over the duration of the AE waveform packet. It is sometimes referred to as relative energy which relates to the energy amount released by the material or structure. Signal strength is independent from gain and calculated over the whole AE signal dynamic range. Thus, signal strength could be one of the more reliable parameters to evaluate the damage level quantitatively compared to other primary parameters such as hit. In fact, AE hit or count cannot provide information about the intensity and strength of signal. Signal strength itself is a function of both the amplitude and duration of the signal. In addition, the total number of hits may be affected by duration of loading and unloading. Hence, the cumulative signal strength (CSS) is considered a feasible parameter to be used for assessing damage evolution in a material.

### 3.2. Intensity Analysis (IA)

IA evaluates the structural significance of an AE event as well as the level of deterioration of a structure by calculating two values called the historic index (HI) and severity (Sr) [28–30]. The HI compares the signal strength of the most recent emissions to the signal strength of all emissions. This requires estimating the slope changes of the cumulative signal strength (CSS) plotted as a function of time. The presence of one or more peaks may reveal the occurrence of new damage or the propagation of damage, respectively.

Severity is the average of *J*, the largest signal strength emissions received at a sensor. As the severity is a measure of structural damage, an increase in severity often corresponds to new structural damage. Analytically, the HI and the Sr are defined as

(1) $$\begin{array}{c} \text{HI}=\frac{N}{N-K}\left(\frac{\sum_{i=k+1}^{N}S_{\text{oi}}}{\sum_{i=1}^{N}S_{\text{oi}}}\right),\\ \text{Sr}=\frac{1}{J}\left(\overset{J}{\underset{m=1}{\sum }}S_{\text{om}}\right), \end{array}$$

where *N* is number of AE emissions (referred to as “hits”) up to time *t*; *S*_oi_ is the signal strength of the *i*th event; *K* and *J* are empirical constants based on the material under investigation [28]. In the present paper the following values for *K* and *J* are used: for *N* ≤ 100, *K* is not applicable; for 101 < *N* < 500, *K* = 0.8*N*; and for *N* > 500, *K* = *N* − 100; for *N* ≤ 20, *J* is not applicable, whereas, for *N* > 20, *J* = 20.

This technique is assessed based on the AE signal strength data collected from each sensor. The maximum values for Sr and HI are normally plotted as intensity chart zones, which indicate the structural significance of the emission.

### 3.3. *b*-Value Analysis

The *b*-value is a parameter defined originally by Gutenberg and Richter for estimating the likelihood of occurrence of earthquakes above a specified magnitude *M* [31]. The equation is as follows:

(2) $$\begin{array}{c} \log_{10}N=a-bM, \end{array}$$

where *M* is the Richter magnitude of earthquakes, *N* is the incremental frequency (i.e., the number of earthquakes with magnitude greater than *M*), *a* is an empirical constant and *b* (which is called *b*-value) is the slope, and *A*_dB_ is the peak amplitude of the AE signal in decibels. The Richter magnitude *M* is proportional to the logarithm of the maximum amplitude (*A*_max⁡_) recorded in a seismic trace and also to the logarithm of the source rupture area (*S*). Equation (2) has been found to be valid for the AE data also after applying the necessary corrections. The correction factor is 20, and it arises due to the fact that the AE peak amplitude is measured in dB, whereas the Richter magnitude of earthquake is defined in terms of the logarithm of maximum amplitude. For the application in AE technique, the Gutenberg-Richter formula, therefore, is modified as follows:

(3) $$\begin{array}{c} \log N=a-b\left(\frac{A_{\text{dB}}}{20}\right), \end{array}$$

where *N* is the incremental frequency (i.e., the number of AE hits or events with amplitude greater than the threshold *A*_*T*_), *a* is an empirical constant, and *b* is the *b*-value [31, 32].

Numerous investigations have been carried out on the diagnosis of concrete and masonry structures by *b*-value analysis [33, 34]. It was stated by Colombo et al. [35] that larger *b*-values represent the stage of microcrack occurrence and as damage progresses a general decreasing trend in *b*-value occurs. The denominator of 20 is a scale factor for the calculation of AE amplitude in dB, while, in the original Gutenberg-Richter equation, the earthquake magnitude is proportional to the logarithm of the maximum amplitude. The factor of 20 is typically applied so that the estimated values are in the range of *b*-values observed from earthquakes, that is, this is only a matter of scaling and nothing more. The *b*-value analysis of the AE signals is, in general, applied to a certain number *n* of AE signals. Suggested values of *n* found in the literature range from 50 to 100. In an independent study, Farhidzadeh et al. [36] showed that there are no significant differences in *b*-value results by taking different values of *n*, ranging from 50 to 100. There are copious studies demonstrating that during the fracture process of concrete structures, a relation can be made between types of cracks and evolution of the *b*-value. Mostly, the *b*-value may increase in the early stage of fracture where microcracks are forming and decrease when macrocracks are being localized [35, 36].

Although significant research has presented the applications of *b*-value, few if any such approaches have been applied for torsion damage quantification. In this study, a *b*-value analysis has been conducted to quantify damage process in large scale RC beams subjected to pure torsion loading. In addition, a Gaussian smoothening filter was implemented to deal with unsmoothed large data sets of the *b*-value which had caused an unclear trend in raw *b*-value preliminary results.

### 3.4. Gaussian Smoothening Filter

Gaussian filters can be used to smooth a signal prior to extracting primary features. Although there are several works that implemented Gaussian filters in signal processing, Farhidzadeh et al. [36] were the first to use this filtering method for AE signals to propose sifted *b*-value (Sb) analysis. It is noteworthy that there are some properties in Gaussian functions which have made them useful in smoothing filters. These features can be listed as follows: (1) the Gaussian function is symmetric about the mean, and the weights assigned to signal values decrease gradually with distance from the mean; (2) the width of the Gaussian function is determined by its spread parameter, that is, the standard deviation. As the standard deviation decreases, the Gaussian function does less smoothing; on the contrary, as the spread parameter increases, the amount of smoothing is increased. (3) The local extrema (e.g., *b*-value drop caused by overloads) observed at one standard deviation is also observable at the smaller standard deviations and no additional local extrema are created as the spread parameter increases [36]. This property plays a key role in the analysis of the change of local extrema across different spread parameters. Generally, Gaussian smoothing is the convolution of a Gaussian window and a 1-D vector of data. The Gaussian smoothing *F*(*x*) of a signal, *f*(*x*), is defined as

(4) F(x)=f(x)∗g(x,σ)=∫−∞∞f(μ)g(x−μ,σ)dμ=∫−∞∞f(μ)(12πσ)exp⁡[−(x−μ)22(σ2)]dμ,

where “∗” denotes convolution with respect to *x*, *g*(*x*, *σ*) is the Gaussian function with the standard deviation *σ*, and *μ* is a dummy variable. These filtering parameters, other than window span, should be well chosen to show the correct and clear trend of *b*-value.

## 4. Experimental Program

### 4.1. Materials and Specimens

**Figure 2:**
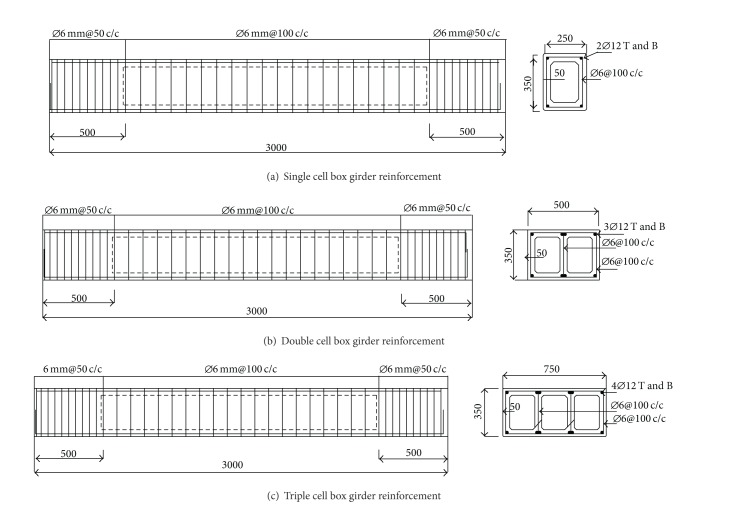
Geometrical detail and reinforcement of girder specimens. (a) Single cell box, (b) double cell box, and (c) triple cell box.

The experimental work consisted of testing three RC box girder specimens under torsion.  Figure 2 shows the geometries and reinforcement details of the specimens. All the specimens have a uniform length of 3000 mm and accommodated single (SC), double (DC), and triple (TC) box cells, respectively.

In RC beams subjected to torsion, cover spalling occurs due to excessive axial thrust on concrete at regions close to beam edges. In order to prevent cover spalling the thickness of the concrete cover should not exceed 30% of *A*_*c*_/*P*_*c*_, where *A*_*c*_ and *P*_*c*_ are the area and perimeter of concrete cross-section, respectively [37]. Therefore all the reinforced specimens prepared with constant concrete cover of 20 mm. The cell wall thickness was fixed at 50 mm, which fulfilled the requirement in resisting axial thrust using the modified softened truss model theory (MSTMT). The wall interior corners were provided with haunches for increased stiffness. In order to apply torque at the end of the specimen, the cross-section of the ends was prepared as solid (fully-filled) rectangular with length of 500 mm. This configuration resembled the fixed connection of the practical support condition, with which the girders were supported rigidly at the ends by columns and transverse beams. In order to obtain the hollow section inside the specimens, polystyrene blocks were placed into the specimen mould before casting with their edges sliced accordingly to provide interior hunches of 30 mm long at four corners of each cell cross-section. After demolding the specimens were covered with dampen sponge sheets and cured for 28 days.

Table 1 gives the mix proportion of the concrete used. Concrete with a designed 28-day compressive strength of 40 MPa was used preparing all the specimens. The mix design complied to (BS 5328:1997) specifications which adopted ordinary Portland cement and coarse aggregates with maximum size of 10 mm. Ready mix concrete with designed compressive strength of 40 MPa was used for all beams. The steel bars used, on the other hand, were of type hot roll deformed bars which gave characteristic strengths of 460 N/mm^2^ and 250 N/mm^2^, for main bars and shear links, respectively.

The mechanical properties of concrete tested at 28 days after casting are shown in Table 2.

### 4.2. Torsion Test

**Figure 3:**
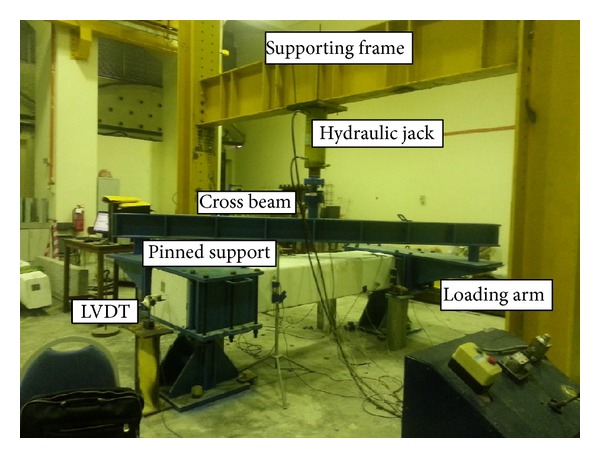
Test machine setup and instruments.

Loading was conducted by a hydraulic actuator setup as shown in Figure 3. A load cell with 450 kN capacity (Interface 1232 AJ-4550 kN-B) was connected to the actuator to measure loading values. The specimens were simply supported by steel rollers with clear span of 2000 mm. Two specially fabricated steel frames with rotating hinges were fixed to both ends of specimens to provide rigid grips. The vertical load applied to the center of a steel spreader beam would be transferred to the steel frame and specimen ends, causing rotation of steel frames about the hinges and induce torsion loading to the specimens. The specimens were tested under monotonically increased torque up to failure. The lateral displacements were measured using LVDTs (liner variable differentiable transformers). The angle of twist was calculated based on measurements of two installed LVDTs at the top and bottom of the specimen. The specimen elongation was found using two LVDTs fixed at center of specimen cross-section at both ends.

### 4.3. AE Monitoring

**Figure 4:**
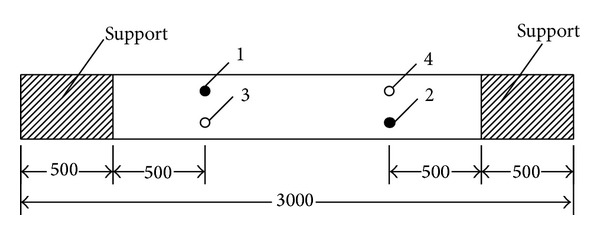
The arrangement of AE sensors mounted on surface of two sides of girders.

The AE measurement system (by MISTRAS Group Inc.) consists of PCI-2 data acquisition controller boards as well as a Windows-based acquisition operating program known as AEWIN. The AE sensors used in this experimental study have resonant frequency of 60 kHz. Four AE sensors were mounted on the specimens as illustrated in Figure 4. Sensors 1 and 2 were mounted on one side surface of specimen with 500 mm distance from the supports at both ends. Sensors 3 and 4 were mounted on the opposite side surface in an “upside down” arrangement relative to sensors 1 and 2, respectively. In the monitoring, the data sampling rate was set at 2 MHz, with the pretrigger set as 250.000 *μ*s and data length at 2 k. To eliminate electrical and mechanical noises, the threshold level was set at 50 dB.

## 5. Test Result and Discussion

### 5.1. Crack Development

**Figure 5:**
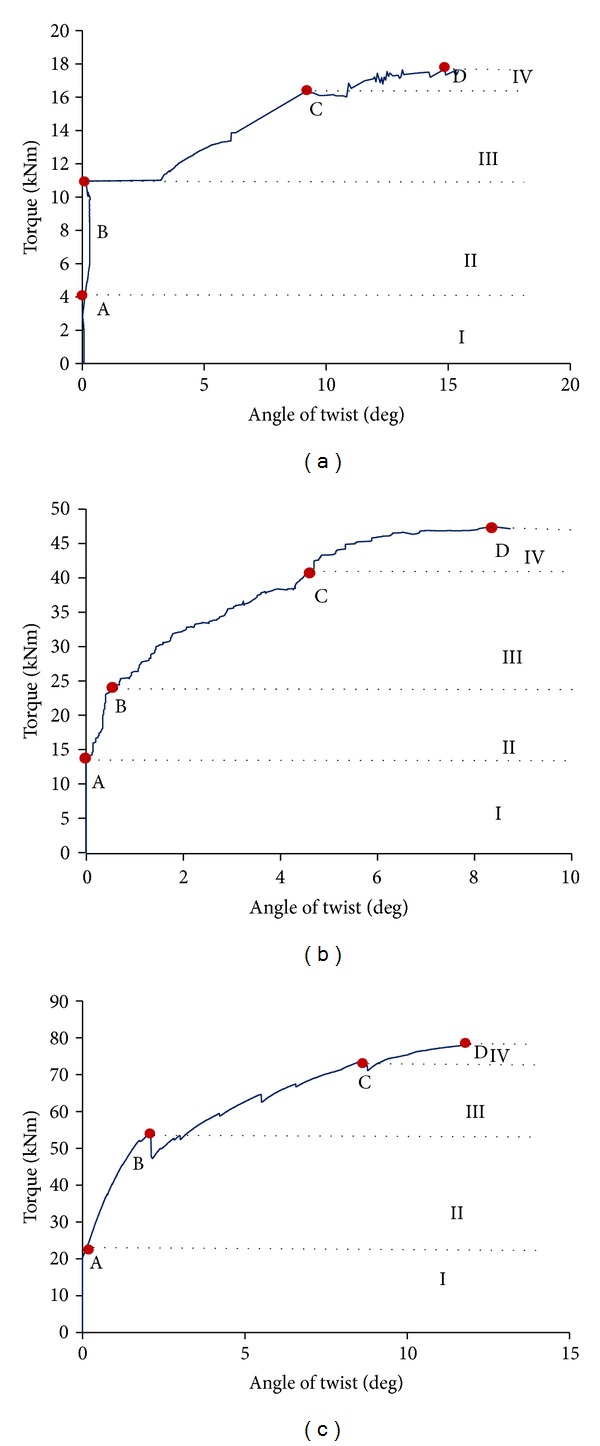
Torque angle of twist: (a) single cell section girder, (b) double cell section girder, (c) and triple cell section girder.

Based on the visual observation on the mechanical fracture behavior, the propagation of crack could be classified into four different stages, namely, microcracking, first visible crack initiation, crack distribution, and damage localization.  Figure 5 shows the relationship between torque and angle of twist of all specimens. It could be observed that in general the twist developed linearly with torque at initial stage of loading. In SC specimen, the first visible crack was observed at 4 kN (Point A in Figure 5(a)). While for DC and TC specimens, it was observed at 14.2 kN and 20 kN (Figures 5(b) and 5(c)), respectively. Prior to this microcracking was considered to have taken place within the specimens, whose occurrence could be detected with the aid of AE monitoring. It was also observed that the location of the first visible crack was near to the midspan of specimen. As the loading was increased, the crack further propagated to cross the side face of the specimen and continued to the top face, while there were some new cracks developing simultaneously. This stage, known as the crack distribution stage, was detected when load reached approximately 50% of its ultimate load (point B) as depicted in Figures 5(a)–5(c). As the increase in loading persisted, cracks propagated in a spiral pattern on the four faces of specimens. This process was observed till the torsional moment reached approximately 70% of the ultimate load, that is, 16.2 kN, 38.5 kN, and 72 kN for SC, DC, and TC specimens, respectively (point C in Figures 5(a)–5(c)). During this stage, the width of the cracks expanded and no new crack initiation was observed, signifying the development of localized fracture by the existing cracks. By considering the definition of stiffness, Figure 5 shows that as load increases the slope of torque-twist curve decreased which implies that the torsional stiffness of the specimens decrease with the widening of the cracks at midspan. The failure mode for all specimens was crushing of concrete.

The ultimate torque and angle of twist of the specimens at failure (point D) are tabulated in Table 3. The ultimate load for DC and TC specimen is 2.7 times and 4.5 times higher than ultimate load of SC specimen, respectively. It can be concluded that the torque based on a single cell assumption is 25% higher than the total torque in case of a double cell based on multicell assumption. For triple cells, the torque based on a single cell assumption is 33% higher than the total torque based on multicell assumption. Apparently, SC specimen has more ductility than the other two specimens DC and TC after reaching peak point at 15.40 degree. The angle of twist for single cell is 1.7 times and 1.3 times higher than that for double and triple cells, respectively.

### 5.2. Damage Localization Process

**Figure 6:**
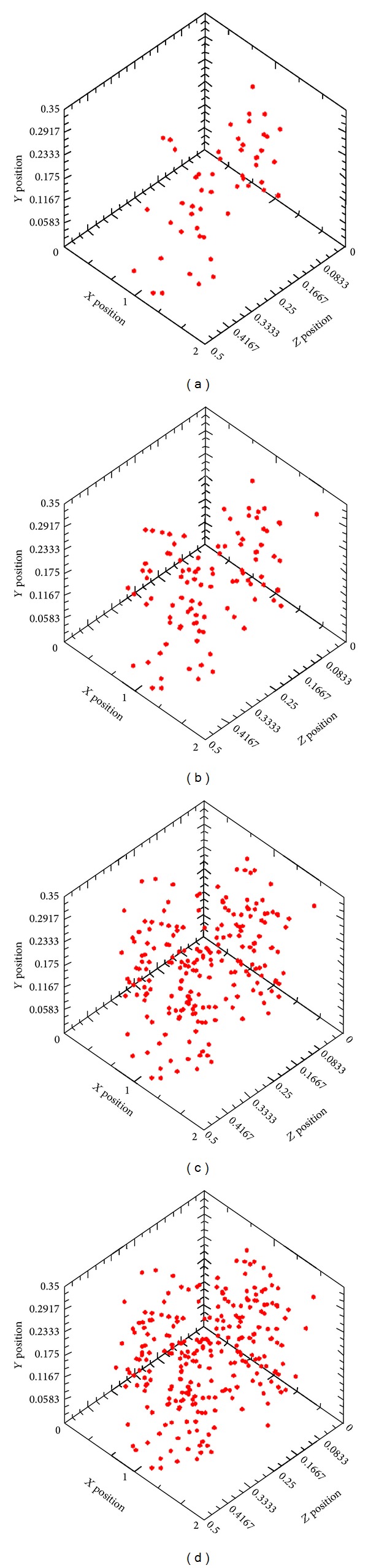
3D even localization of damage progress with respect to progressive damage evolution. (a) Stage I, (b) stage II, (c) stage III, and (d) stage IV.

Localization technique is required for quantitative methods in acoustic emission analysis in order to accurately obtain the source coordinates of the AE events. In practice, there is a variety of ways for AE localization to obtain the required resolution in one, two, or three dimensions. The principal of acoustic emission source localization arose from modified earthquake source localization. A detailed description of earthquake localization methods can be found in [4]. To determine the source location, the differences in the arrival times of recorded elastic waves emitted by the fracture at each sensor should be inversely calculated. The source location of acoustic emission is defined by the origin time (start of the rupture) and the source position in Cartesian coordinates (*x*0, *y*0, and *z*0). The onset time of the compression wave (*P*-wave) is the first arrival time of the elastic wave at each sensor [4]. In this study a 3D localization system has been conducted where the principal is quite similar to the determination of earthquake hypocenters in seismology and uses the recorded arrival waves of an earthquake at multiple seismometers. These algorithms can be modified to fit material testing requirements and afford the possibility to study different specimen geometries in addition to considering the number of transducers and their arrangement around the object.  Figure 6 shows the crack and damage progress in the specimens under pure torsion loading.

It can be conferred from the figures that, although there are only four sensors mounted on the specimens, there was still a possibility to monitor the active damage under the loading condition. First, it can be observed that in early stage of damage, there are a few events which have occurred in the area nearby position of S1 and S4. The pattern of the occurrence of the event due to progress of damage from early stage (microcrack) to macrocrack stage tracks the pattern of events in early stage. This pattern also adapts the pattern of occurrence of the crack which was propagated in a spiral direction throughout the beam. It is demonstrated that the specimens under the pure torsion will undergo the inclined cracks which propagate in spiral direction. As it was observed in the early stage there were a few events, while the rate of occurrence of AE signals rose in the second and third stage of damage, especially in the transition of first visible crack to the stage of crack localization. On the other hand, in the last stage while the cracks are being widened rather than being propagated, again the rate of occurrence of AE events dropped. This phenomenon also conforms to the *b*-value results in another way; that is, when in the early stages the number of occurrence of AE events is high the amplitude of AE signals is lower, whereas in the last stage, there are fewer numbers of AE event with higher amplitude.

### 5.3. Qualitative AE Analysis

#### 5.3.1. Cumulative AE Hits

**Figure 7:**
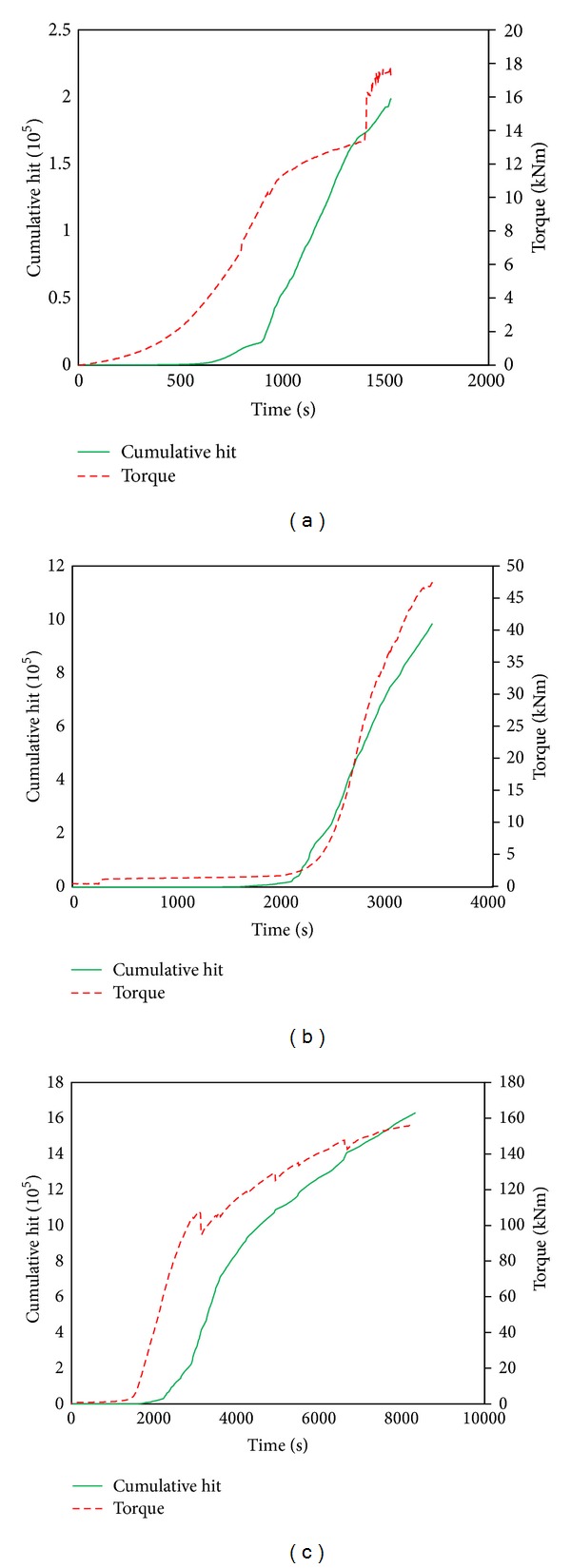
Cumulative hits for all three girders, the plot of torque moment is imposed. (a) Single cell section girder, (b) double cell section girder, and (c) triple cell section girder.

The relation between torque time of loading and AE hits data of the specimens is given in Figure 7. The torque moment applied on specimens has caused rotation and microcrack initiation. The transient release of strain energy as a result of microcracking was detected by AE sensors and identified as an AE hit. During the initial stage of loading, the cumulative AE hit was low until occurrence of microcracks. As damage in the specimens intensified, macrocracking took place and by the time the first visible crack was found the cumulative AE hit increased significantly. In the case of TC specimen, the microcracking stared 10 seconds into the test (triggering point) and there was no significant increase in AE hit till the first visible cracking. Thereafter, the rate of cumulated AE hits increased as the damage continued to progress until ultimate failure.

#### 5.3.2. AE Signal Strength Analysis

**Figure 8:**
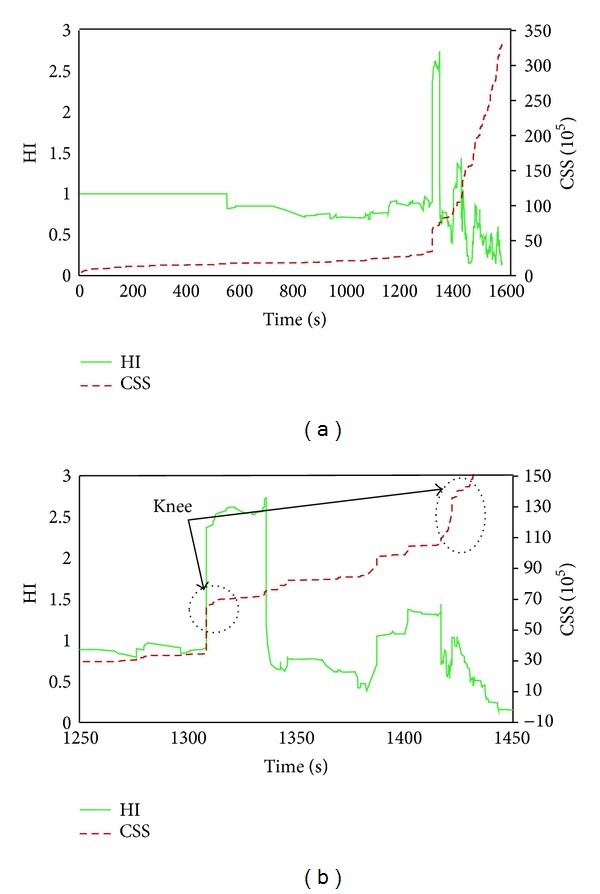
AE signal strength analysis for DC specimen. (a) Historic index as a function of time. The plot of the cumulative signal is superimposed. (b) Magnified portion of plot (a).

**Figure 9:**
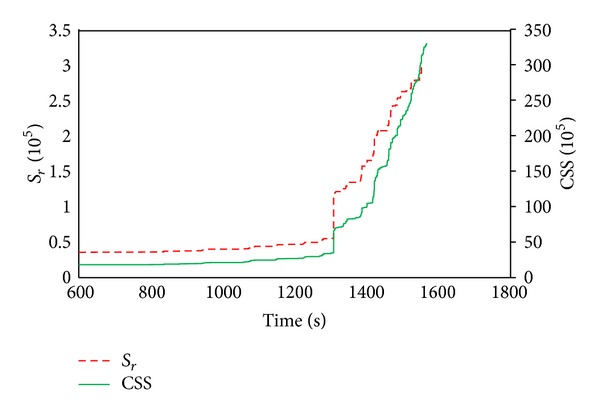
Severity index as a function of time for DC specimen. The plot of the cumulative signal is superimposed.

Signal strength is a function of both amplitude and duration of signal. The relationship between cumulative signal strength and historic index could be utilized as a reliable parameter for evaluating damage in structures qualitatively. The condition of specimen can be assessed with data given in Figure 8, in which the historic index (HI) and cumulative signal strength (CSS) were plotted and shown as functions of time. For clarity, a magnified portion of Figure 8(a) is depicted in Figure 8(b). The presence of an AE “knee” defined as a point of significant change in the slope of the CSS was highlighted in the figure. The AE knee may be used to identify possible damage mechanisms and to locate the onset of failure [29]. At loading time of 1200–1260 s, AE knee was obtained for DC specimen. The time interval was in correspondence to the occurrence of the first visual crack, signifying the change from micro- to macrocracking. Concurrently, high density peaks were observed in the data curve of HI. With increase of torque, the CSS data curve increased gradually as a sign of stage crack propagation. The second AE knee was noticed on CSS data curve at time between 1440 s and 1500 s, which was related to the yielding of stirrups in the midspan of DC specimen. Prior to the initiation of the first visible crack, some HI peaks were observed on data curve. The values of these peaks were not substantial and were corresponding to initiation of microcracks within the specimen.  Figure 9 shows the severity (Sr), as a function of time with a plot of SCC superimposed. Both plots were qualitatively very similar. Distinct changes could be observed in both Sr and CSS curves at almost the same time as indication to the apparent increase of damage in the specimen under loading. It has been demonstrated that signal strength as a conventional AE parameter, either in the form of cumulated or uncumulated values, can be taken as an indication of damage state in specimen. As can be seen in Figure 9 signal strength variation (either SCC or Sr) was negligible upon visible cracking point at which sudden increase occurred in signal strength. This incremental trend might indicate changes in damage state from stage II to stage III. A second incremental change was in correspondence to yielding of stirrup. There after a gradual increasing trend implies macrocrack opening width which is a progressing damage till final failure.

### 5.4. Quantitative AE Analysis

#### 5.4.1. Intensity Analysis (IA)

**Figure 10:**
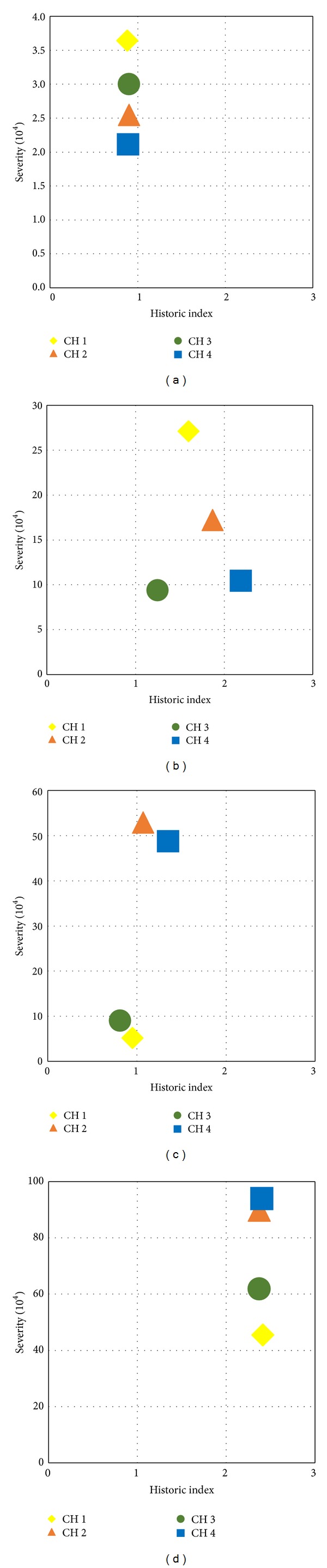
Intensity chart for double cell specimen at each stage of damage. (a) Stage I, (b) stage II, (c) stage III, and (d) stage IV.

By adopting IA technique, the acquired AE data was used to obtain the indices from (1). The intensity chart was prepared by plotting the maximum of the average values of HI and Sr for each stage of damage.  Figure 10 shows the intensity charts for double cell specimen. The maximum values of HI and Sr from each channel obtained for each stage of damage in the current experiment are plotted in Figure 10. In general, intensity values that are clustered toward the top are associated with the phenomena of high structural significance events, whereas less structurally significance events could be found concentrating near the bottom of the chart [37].

Figure 10(a) shows the intensity chart for DC specimen at first stage of damage (stage I). From the start of loading and prior to initiation of the first surface crack, no significant AE activity was recorded with all channels. Therefore based on the IA, the specimen condition could be considered to be in stage, recognized as the microcracking stage. As load was increased and the first visible crack was detected on the face of specimen, significant increase in severity index value was observed at channel 1 which was closer to the area of first visible cracking. The damage condition shifted to stage II, which represented the minor surface cracking. After occurrence of the first visible crack, the transverse strain of stirrups was found to increase dramatically. Crack propagation and distribution was observed more at left side of the specimen where sensors 2 and 4 were mounted. The higher Sr values were obtained at channels 2 and 4, which confirm that the greater amount of damage occurred in the area of channels 2 and 4. In stage III of damage elaboration of the defects is required. As load was increased until the maximum, cracks started to localize to form major cracks, with the width of cracks increasing considerably. The value of severity index from all channels was increased considerably. The damage severity stage was identified to be in stage (IV) which was known as serious fracture that required detailed inspection.

#### 5.4.2. Damage Quantification by *b*-Value Analysis

**Figure 11:**
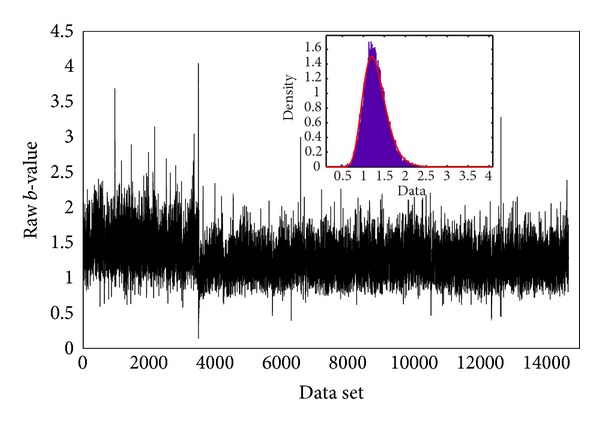
*b*-values results for the entire data set before smoothing process for TC specimen.

**Figure 12:**
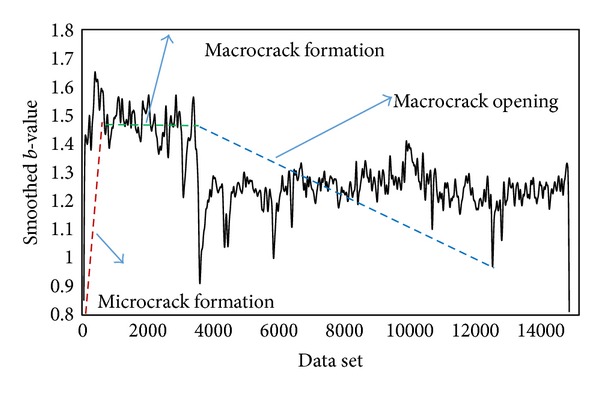
Smoothed *b*-values results for entire data set of TC specimen.

A *b*-value analysis was performed to quantify torsion damage of specimens in a different way. The *b*-value was computed based on using groups of *n* = 100 AE signals.  Figure 11 illustrates the *b*-value obtained taking into account AE signals recorded from the whole monitoring period for TC specimen. The estimated probability density function is placed in the top side of Figure 11. It is interesting to find that a Gaussian distribution could be fitted reasonably well with the mean and the standard deviation calculated from the *b*-values. As can be seen for the figure, although the general decreasing trend of the *b*-value was indicative of the evolution and progress of damage in the specimen, it became challenging to identify a definite trend for the *b*-value in each particular stage of damage. The discrepancy that occurred most likely attributed to the large amount of AE activities generated during the process concrete fracturing during torsion loading. In the case for the other specimens, similar results were obtained for the DC specimen but not for the SC specimen because of the insufficient data for analysis. It is reckoned that the difficulty in identifying a proper *b*-value trend for each stage of damage could be a drawback in real time monitoring of structures. Therefore, to provide a reliable elaboration for *b*-value trends associated with the progress of damage, the Gaussian filtering was introduced to analysis. The window span selected was 5% of the data vector length. In addition, the standard deviation was set as a quarter of the window span.  Figure 12 shows the smoothed *b*-value data after application of Gaussian filtering method. The corresponding damage level is indicated in the graph. As can be seen, from the figure, after filtering the raw data, the trend of *b*-value variation corresponding to each stage of damage could be distinguished more confidently. Three distinct stages of damage were recognized to have influenced the trend of *b*-value. In general, the *b*-value exhibited during stage I damage was pertinent to initiation and accumulation of microcracks. As fracture in concrete shifted to stage II, which was associated with the formation of the first visible crack followed by macrocracking, slight fluctuation in *b*-values results was registered, accompanied by a drop in magnitude. Finally, in stage III of damage, which is also called macrocrack, a significant drop occurred, while thereafter general decrease is imposed on *b*-value. It should be noted that the fluctuation seen in stage II could be associated with formation of a few microcracks that happened in this stage, while the dominant cracks are macrocracks.

The *b*-value could be regarded as a useful indicator to the stage of damage.  Table 4 gives the *b*-value results for all specimens during the different levels of fracture under torsion. It was found that the evolution of damage in RC box girder specimens due to torsional loading could cause significant drop in the *b*-value. The maximum drop for as acquired from the current analysis ranged between 70% and 75%. It could thus be inferred that the drop in *b*-value within this range was an indication of critical fracture developed in girders under torsion. A gradual decrease for *b*-value from stage I to stage III of damage was noteworthy as well, with the *b*-value decreasing to approximately 1.00 before ultimate failure.

## 6. Conclusion

Results of AE monitoring of reinforced multicell hollow girder specimens are presented in this paper. The AE technique was implemented to characterize torsional failure of girder specimens. AE analysis were carried out in order to provide fundamental information for damage assessment of girder specimens, both qualitative and quantitatively. Hits and signal strength were obtained to provide qualitative evaluation of damage process of girders under monotonic load. Correlation between torque curves and AE cumulative hits for specimens was in good agreement. Analysis of CSS slopes demonstrated that when significant structural events such as macrocracking and rebar yielding occur, CSS experiences significant changes in the form of a knee in its curve. This study indicates that cumulative hits and signal strength correlate well with the degree of damage of specimens. Furthermore intensity chart was generated for the DC specimen up to the failure point. The results obtained from intensity chart demonstrate the efficiency of this analysis to provide quantitative information on the level of damage severity. In addition, *b*-values results can be used as a useful quantitative indication of damage. In general, *b*-value may increase in the early stage of fracture where microcracks are forming and decreases when macrocracks are being localized. When the average of *b*-values results are approaching unity that might be an indication of serious damage or final failure of the structure subjected to torsion loading.

To sum up, both qualitative and quantitative methods might be required to assess the level of damage and register the true pattern of the structure failure.

## 7. Future Study

It should be noted that the evolution of the *b*-value could be interpreted according to the physical dimension of the damaging domain (usually noninteger), emphasizing the transition from diffused microcracking to localized macrocracks. Hence, as a future study this can be extensively examined. In addition, although there are a few studies which used *b*-value analysis for monitoring full scale structures [6, 31], the effect of scaling has not been extensively studied. Since the *b*-value evolution is associated with variation of amplitude, it might be affected by the effect of signal attenuation for large scale structures. Therefore, there are many features of utilizing *b*-value to be studied further for providing a reliable quantitative monitoring parameter.

## Figures and Tables

**Table 1:** Concrete mix proportion.

Material	Fine aggregate (kg/m^3^)	Coarse aggregate (kg/m^3^)	W/C ratio	Super plasticizer, R1100 (liter/m^3^)	Water reducer, P218R (liter/m^3^)
	890	800	0.42	3.6	1.0

**Table 2:** Mechanical properties of concrete after 28 days of curing.

Compressive strength (MPa)	Splitting tensile strength (MPa)	Modulus of elasticity (GPa)
45.06	2.75	31.8

**Table 3:** Ultimate torque and corresponding twist angle of specimens.

Specimens	Ultimate torque, (kNm)	Twist at ultimate torque (o)
SC	17.5	15.4
DC	47.13	8.9
TC	78.3	11.9

**Table 4:** The average *b*-values for each stage of damage.

Specimen	*b*1	*b*2	*b*3	∗Maximum drop
SC	1.44	1.30	1.05	73%
DC	1.50	1.32	1.12	75%
TC	1.68	1.41	1.16	70%

∗This value presents drop of *b*-value in last stage relative to the first stage.
